# Risk factors and incidence of new-onset heart failure with conventional pacemaker implant: A nationwide study

**DOI:** 10.1016/j.hroo.2024.07.012

**Published:** 2024-07-23

**Authors:** Maiwand Farouq, Cecilia Rorsman, Sofia Marinko, David Mörtsell, Uzma Chaudhry, Lingwei Wang, Pyotr Platonov, Rasmus Borgquist

**Affiliations:** ∗ Cardiology Section, Department of Clinical sciences, Lund University, Lund, Sweden; † Arrhythmia Section, Skane University Hospital, Lund, Sweden; ‡ Internal Medicine Department, Varberg Hospital, Varberg, Sweden

**Keywords:** Heart failure, Pacemaker, Prognosis, Register, Mortality, Resynchronization therapy, Risk score

## Abstract

**Background:**

Studies have shown that the risk of new-onset heart failure (HF) is higher postimplantation for patients receiving right ventricular pacing.

**Objective:**

This study aimed to investigate incidence, risk factors, and implications for long-term prognosis of new-onset HF in patients after pacemaker implantation.

**Methods:**

Patients without pre-existing HF who received a pacemaker in Sweden during the period of 2005 to 2020 were identified via the nationwide Pacemaker Registry. Data were crossmatched with the population registry and national disease registries. The primary outcome was new-onset HF within 5 years, and a risk score for this was developed and validated.

**Results:**

In all, 65,579 patients met the inclusion criteria (10,351 single-chamber ventricular and 55,228 dual-chamber pacemakers). A total of 13,792 (21.0%) patients were diagnosed with HF within 5 years postimplantation. Of these, 6244 (45.3%) were hospitalized for HF. Patients with new-onset HF were more likely to die within 5 years (41.2% vs 19.7%, *P* < .0001). Risk factors for new-onset HF included increasing age, male sex, hypertension, diabetes, atrial fibrillation, chronic lung and kidney disease, ischemic heart disease, and atrioventricular block. In a combined score using these variables, patients in the highest risk-score quartile had a hazard ratio of 5.36 (95% CI 4.91–5.86, *P* < .001) and an absolute risk of 32% for developing HF.

**Conclusion:**

Pacemaker therapy is associated with >20% risk of new-onset HF within 5 years, and we identified 9 risk factors associated with the diagnosis of new-onset HF. The proposed score based on these variables can be used to identify patients at high risk for new-onset HF.

## Graphical abstract

**Figure:**
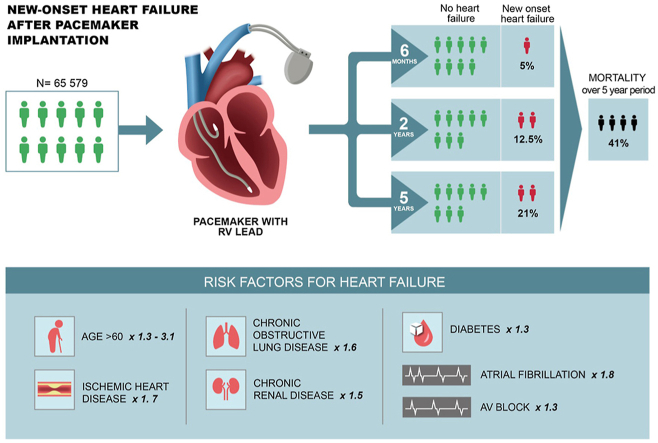

**Key Findings**

▪ In this nationwide cohort, the risk of new-onset heart failure after pacemaker implantation was more than 20% within the first 5 years.
▪ Using the proposed risk factor score may help to identify patients with high risk of heart failure already prior to implantation.
▪ Randomized studies are needed to determine if other pacing modalities would be a better choice for patients with high risk for heart failure and in need of antibradycardia pacing.

## Introduction

There is a known association between right ventricular (RV) pacing and development of heart failure (HF), and in the newest version of European Society of Cardiology guidelines on cardiac pacing, the risk of pacemaker-induced cardiomyopathy is highlighted.^1^ While cardiac resynchronization therapy (CRT) has been demonstrated to be superior compared with RV pacing in patients with AV block and symptomatic HF,^2^ patients without prior HF who are subjected to RV pacing may have up to 20% risk of developing HF postimplantation.^3^, ^4^, ^5^ A Danish registry-based study used an age-matched control cohort from the general population to evaluate adjusted incidence of new-onset HF after pacemaker implantation and concluded that there was a more than 10% higher risk for HF in the pacemaker-treated population.^6^ A similar study from the United States found the incidence of new-onset HF post–pacemaker implantation to be >10% within 6 months, and to increase with higher age.^7^ The rationale is that RV pacing induces an altered activation pattern, and in patients with previously narrow QRS complexes, this can induce dyssynchrony in a similar fashion to a native left bundle branch block, with late activation of the lateral parts of the left ventricle.^8^ With a significant amount of RV pacing, the dyssynchrony can in turn lead to left ventricular remodeling and secondary HF, or aggravated HF if the patient had HF already prior to pacemaker implantation.^3^^,^^9^ Previous studies have highlighted renal failure, prior myocardial infarction, and atrial fibrillation as risk factors in this context. However, previous studies have typically not had access to all relevant comorbidity variables, and the prognostic significance of new-onset HF remains to be described in a large cohort. Furthermore, an increasing proportion of patients with bradycardia receive leadless pacemakers. The leadless devices have several advantages over transvenous devices, and also the disadvantage that device upgrade to CRT currently is not feasible, which means that it is even more important to identify patients at risk of pacemaker-induced cardiomyopathy prior to device implantation.^10^

We set out to investigate this in a nationwide cohort of all pacemaker-treated patients in Sweden during the period of 2005 to 2020.

## Methods

### Data sources

The nationwide Swedish Pacemaker Register includes all implanting centers in Sweden and covers 97% of all implants. We identified all patients who had received a primary implant of a single- or dual-chamber pacemaker during the period of 2005 to 2020, with a pacing lead in the RV for myocardial pacing. Pacemakers with left ventricular leads (CRT) or conduction system pacing (CSP) leads were excluded. Patients with implantable defibrillators or leadless pacemakers were also excluded. Based on the unique Swedish personal identification number, data were then crossmatched with data from the Swedish Cause of Death Register and the Outpatient and In-patient Health Care Registers for diagnoses and mortality endpoints. Coding of diagnoses was for the entire relevant time-period for this study done using the International Classification of Diseases–Tenth Revision–Swedish Edition classifications. HF was defined as codes I50.∗-I51.∗ or I42.∗-I43.∗. The registers and the Swedish system of crossmatching data using the personal identification number have previously been validated, and the positive predictive value has been above 90%.^11^ The specific diagnosis of HF has also been validated, showing a validity of 95% for HF as the primary diagnosis.^12^ A subset of 822 randomly chosen patients was used to evaluate the effects of key variables that were not available in the registry database. The study was approved by the Swedish Ethical Review Authority (2020-02035 and 2021-05826-01).

### Study endpoints

The primary study endpoint was incidence of new-onset HF within 5 years. In secondary analysis, all-cause mortality within 5 years postimplantation was evaluated, stratified for new-onset HF. For the mortality analysis, the study sample was divided into 2 groups: those who were diagnosed with new-onset HF within 5 years postimplantation and those who were not (control subjects). In prespecified subanalyses, incidence of new-onset HF within 6 months and 2 years postimplantation was also investigated. HF diagnosis within 30 days of the implantation was disregarded unless the diagnosis was repeated at a later time point during the follow-up. The rationale for this was that a first-time diagnosis of HF in conjunction with the index hospitalization (ie, the time of pacemaker implantation) would result in HF diagnosis “after” the implantation date, when in fact this may have occurred prior to implantation and due to congestive symptoms caused by atrioventricular (AV) block and bradycardia. However, if the diagnosis was repeated later during follow-up, it was included in the usual way. The research reported in this study adhered to the Helsinki guidelines. The Swedish Ethical Review Authority waived the requirement of individual patient consent, due to the use of retrospective and de-identified data.

### Statistical methods

Continuous variables (not normally distributed) are presented as median (interquartile range) and compared using the Mann-Whitney *U* test, whereas categorical variables are presented as counts and percentages and compared using Fisher’s exact test or the chi-square test as appropriate. Age was a strong graded predictor for both new-onset HF and all-cause mortality. Patients were therefore divided into 5 decile groups based on their age (<60 years, 60–69 years, 70–79 years, 80–89 years, and ≥90 years), and the age group <60 years was used as reference. When counting risk factors, age above 60 years received 1 point for each incremental decile group.

For construction of the risk score, the dataset was randomly split into 2 subsets, each representing approximately 50% of the total number of patients; a training set (n = 32,578) and a validation set (n = 33,001). The training set was used to identify significant predictors for HF, and to construct a score. The performance of the score was then tested in the validation set. Adjusted logistic regression analysis was used to evaluate risk ratio for incident HF at 5 years. Adjusted Cox regression analysis with new-onset HF as time-dependent covariable (to account for immortal time bias) was used to evaluate the hazard ratio (HR) for survival up to 5 years from time of implantation. The final multivariable models (both logistic regression and Cox regression) were adjusted for baseline variables that were associated with the respective outcomes in univariable analysis (defined by *P* < .05).

In a sensitivity analysis, interceding myocardial infarction was treated as competing risk, and patients with main diagnosis acute myocardial infarction between implantation date and date of new-onset HF diagnosis (n = 1409) were excluded. This did not significantly alter the HRs or significances of the other variables (Supplemental Table 1). Data management was performed in SAS version 9.4M7 (SAS Institute), statistical analyses were performed using SPSS version 27 (IBM).

## Results

**Figure 1:**
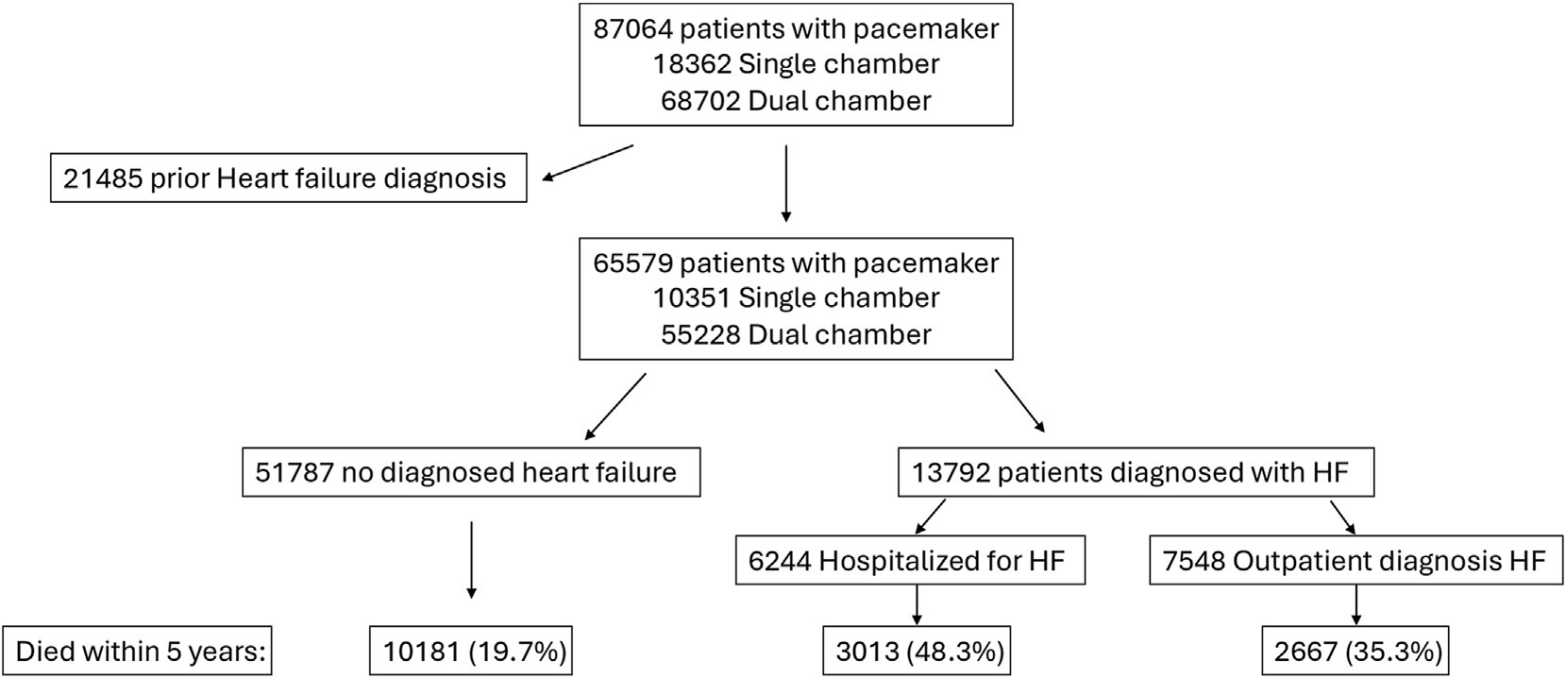
Flowchart over patient inclusion and 5-year mortality for each subgroup. HF = heart failure.

During the period of 2005 to 2020, a total of 87,064 patients with a device capable of pacing the RV were identified from the Pacemaker Register. Of these, 21,485 had a prior diagnosis of HF within 5 years before the implantation date and were excluded (see Figure 1). The remaining 65,579 patients met the inclusion criteria and consisted of 10,351 single-chamber and 55,228 dual-chamber pacemakers. The cohort was split into 2 groups: those who were diagnosed with new-onset HF within 5 years postimplantation and those who were not. Baseline demography is presented in Table 1, and the majority of patients were men (n = 37,785 [57.6%]) and the median age was 77.6 (interquartile range [IQR] 70.0–83.9) years. The total follow-up time was median 5.0 (IQR 2.2–8.5) years, longer for the group with diagnosed HF: 5.1 (IQR 2.1–8.8) years vs 4.6 (IQR 2.7–7.3) years (*P* < .001).

**Table 1:** Baseline demographic data and comorbidities.

	No heart failure (n = 51,787)	Incident heart failure (n = 13,792)	All patients (N= 65,579)
Female	22,093 (42.7)	5701 (41.3)	27,794 (42.4)
Age, y	76.7 (68.9–83.3)	80.6 (74.3–85.8)	77.6 (70.0–83.9)
<60 y	5369 (10.4)	520 (3.8)	5889 (9.0)
60–69 y	9002 (17.4)	1485 (10.8)	1048 (16.0)
70–79 y	18,143 (35.1)	4489 (32.6)	22,632 (34.5)
80–89 y	16,152 (31.2)	6044 (43.9)	22,196 (33.9
≥90 y	3083 (6.0)	1241 (9.0	4324 (6.6)
Cerebrovascular disease	4156 (8.0)	1291 (9.4)	5447 (8.3)
Atrial fibrillation	19,221 (37.3)	7284 (52.8)	26,575 (40.5)
Any ischemic heart disease	11,858 (22.9)	5125 (37.2)	16,983 (25.9)
IHD (chronic)	7878 (15.2)	3528 (25.6)	11,406 (17.4)
Prior AMI	3912 (7.6)	1874 (13.6)	5786 (8.8)
Angina pectoris	6109 (11.8)	2672 (19.4)	8781 (13.4)
Hypertension	28,436 (54.9)	8729 (63.3)	37,165 (56.7)
Malignancy	12,700 (24.5)	3785 (27.4)	16,485 (25.1)
Diabetes	8205 (15.9)	3053 (22.1)	11,303 (17.2)
Type 2	7976 (15.4)	2947 (21.4)	10,923 (16.7)
Type 1	1446 (2.8)	680 (4.9)	2126 (3.2)
Chronic liver disease	125 (0.2)	30 (0.2)	155 (0.2)
Chronic kidney disease	1474 (2.8)	742 (5.4)	2216 (3.4)
Sarcoidosis	151 (0.3)	61 (0.4)	212 (0.3)
COLD	2039 (3.9)	951 (6.9)	2990 (4.6)
ECG indication			
Normal rhythm	440 (0.8)	85 (0.6)	525 (0.8)
AV block	24,313 (47.0)	5981 (43.4)	30,294 (46.2)
BBB or fascicular block	2291 (4.4)	531 (3.9)	2822 (4.3)
Atrial fibrillation	6394 (12.4)	3050 (22.1)	9444 (14.4)
Sick sinus syndrome	18,327 (35.4)	4141 (30.0)	22,468 (34.3)

Values are n (%) or mean (range).

AMI = acute myocardial infarction; AV = atrioventricular; BBB = bundle branch block; COLD = chronic obstructive lung disease; ECG = electrocardiography; IHD = ischemic heart disease.

In all, 13,792 (21.0%) patients were diagnosed with HF within 5 years postimplantation. Of these, 6244 (45.3%) were also hospitalized for HF within the same time period. In adjusted Cox regression analysis with incident HF as a time-dependent covariable, patients with new-onset HF were more likely to die within 5 years compared with those without new-onset HF (HR 3.55, 95% confidence interval [CI] 3.43–3.68, *P* < .0001). Absolute 5-year mortality was increased for all patients with new-onset HF and was highest for those who were hospitalized for HF (Figure 1).

Evaluation of % RV pacing was performed by manual review of medical records in a subset of 825 randomly chosen patients: in patients with indication “AV block or atrial fibrillation with slow ventricular rate,” % RV pacing was median 80.5% (IQR 9.7%–99%) after 2 months and 92% (IQR 20%–99.5%) after approximately 2 years. For those with other pacing indications (primarily sick sinus syndrome, but also bundle branch block and paroxysmal atrial fibrillation), corresponding values were 1.6% (IQR 0%–17.3%) and 2% (IQR 0%–21%) (*P* < .0001) for difference between groups on both measurements. Correspondingly, in patients with high-degree AV block or slow atrial fibrillation, QRS duration preimplantation was 104 (IQR 90–140) ms vs 96 (IQR 86–110) ms (*P* < .0001) for those with other pacing indications. Postimplantation, the patients with AV block had a QRS duration of 156 (IQR 104–178) ms vs 106 (IQR 98–138) ms (*P* < .0001). % RV pacing was strongly associated with increased risk of HF; HR per a 10% increase in RV pacing was 1.09 (95% CI 1.06–1.13) (*P* < .00001). Demographic data for the subset cohort are shown in Supplemental Table 2.

For the construction and validation of the risk score, the material was split into a test cohort and a validation cohort, as described in the Methods. Based on analyses on the test cohort, risk factors associated with new-onset HF are presented in Table 2. A combined score using the logarithmic odds ratios from the significant variables in the multivariable model was constructed:

**Table 2:** Risk factors associated with higher risk of new-onset heart failure within 5 years postimplantation, in the test cohort.

	Univariable analysis		Multivariable analysis	
	*P* value	Odds ratio (95% CI)	*P* value	Odds ratio (95% CI)
Male	.01	1.07 (1.01–1.013)	.01	1.08 (1.02–1.14)
Age				
<60 y		Reference		
60–69 y	<.001	1.65 (1.41–1.91)	<.001	1.29 (1.11–1.50)
70–79 y	<.001	2.48 (2.16–2.84)	<.001	1.78 (1.55–2.95)
80–89 y	<.001	3.68 (3.21–4.21)	<.001	2.62 (2.28–3.00)
≥90 y	<.001	4.17 (3.56–4.89)	<.001	3.07 (2.61–3.62)
Cerebrovascular disease (any)	<.001	1.16 (1.06–1.28)	.22	0.94 (0.86–1.04)
Atrial fibrillation (any)	<.001	1.88 (1.78–1.99)	<.001	1.83 (1.73–1.93)
Ischemic heart disease (any)	<.001	1.97 (1.86–2.09)	<.001	1.68 (1.59–1.79)
Hypertension	<.001	1.42 (1.34–1.50)	.015	1.07 (1.01–1.14)
Malignancy	<.001	1.17 (1.10–1.24)		
Diabetes (type 1 or type 2)	<.001	1.48 (1.39–1.58)	<.001	1.32 (1.23–1.42)
Chronic liver disease	.97	1.01 (0.59–1.73)		
Chronic renal disease	<.001	1.96 (1.72–2.23)	<.001	1.50 (1.31–1.72)
Chronic obstructive lung disease	<.001	1.77 (1.58–1.99)	<.001	1.60 (1.42–1.80)
AV block or AF with slow rate	<.001	1.28 (1.21–1.35)	<.001	1.30 (1.23–1.38)

Logistic regression was performed with univariate association between predictor variable and incident heart failure, and then in a multivariable model including all univariable variables with *P* < .05.

AF = atrial fibrillation; AV = atrioventricular; CI = confidence interval.

Risk score = –2.794 + male sex∗0.076 + atrial fibrillation∗0.602 + hypertension∗0.074 + ischemic heart disease∗0.52 + diabetes∗0.278 + chronic renal disease∗0.407 + chronic obstructive lung disease∗0.468 + pacemaker indication AV block or atrial fibrillation with slow rate∗0.262. The score was split up in equal quartiles, with quartile 1 having the lowest score and quartile 4 the highest score. Based on the score, individual risk for new-onset HF could be calculated using the formula 100∗(exp(risk score)/1+(exp(risk score)).

The performance of the score was then evaluated in the validation cohort, and in Table 3 both the odds ratios and the median risk for new-onset HF (for the respective quartiles of risk score points) are presented. In Cox regression analysis, the score was also associated with a higher risk of hospitalization for HF (HR 1.76, 95% CI 1.72–1.80, *P* < .001 per incremental quartile) and a higher 5-year mortality (HR 1.76, 95% CI 1.72–1.80, *P* < .001), with similar HRs.

**TABLE 3:** Risk of new-onset heart failure based on risk score (in the validation cohort).

Score	*P* value	Odds ratio (95% CI)	Risk of heart failure (%)
Quartile 1 (lowest risk score)	Reference	—	10.0 (7.9–12.4)
Quartile 2	<.001	2.08 (1.89–2.28)	16.6 (14.4–17.7)
Quartile 3	<.001	3.26 (2.98–3.57)	22.1 (20.7–23.9)
Quartile 4 (highest risk score)	<.001	5.36 (4.91–5.86)	32.4 (29.0–38.2)

Values are median (interquartile range), unless otherwise indicated. The score includes the variables sex, age, hypertension, atrial fibrillation, ischemic heart disease, diabetes (type 1 or type 2), chronic kidney disease, chronic obstructive lung disease and pacemaker indication atrioventricular block or atrial fibrillation with slow ventricular rate. Absolute risk of new-onset heart failure for each group is presented in a separate column.

CI = confidence interval.

**Figure 2:**
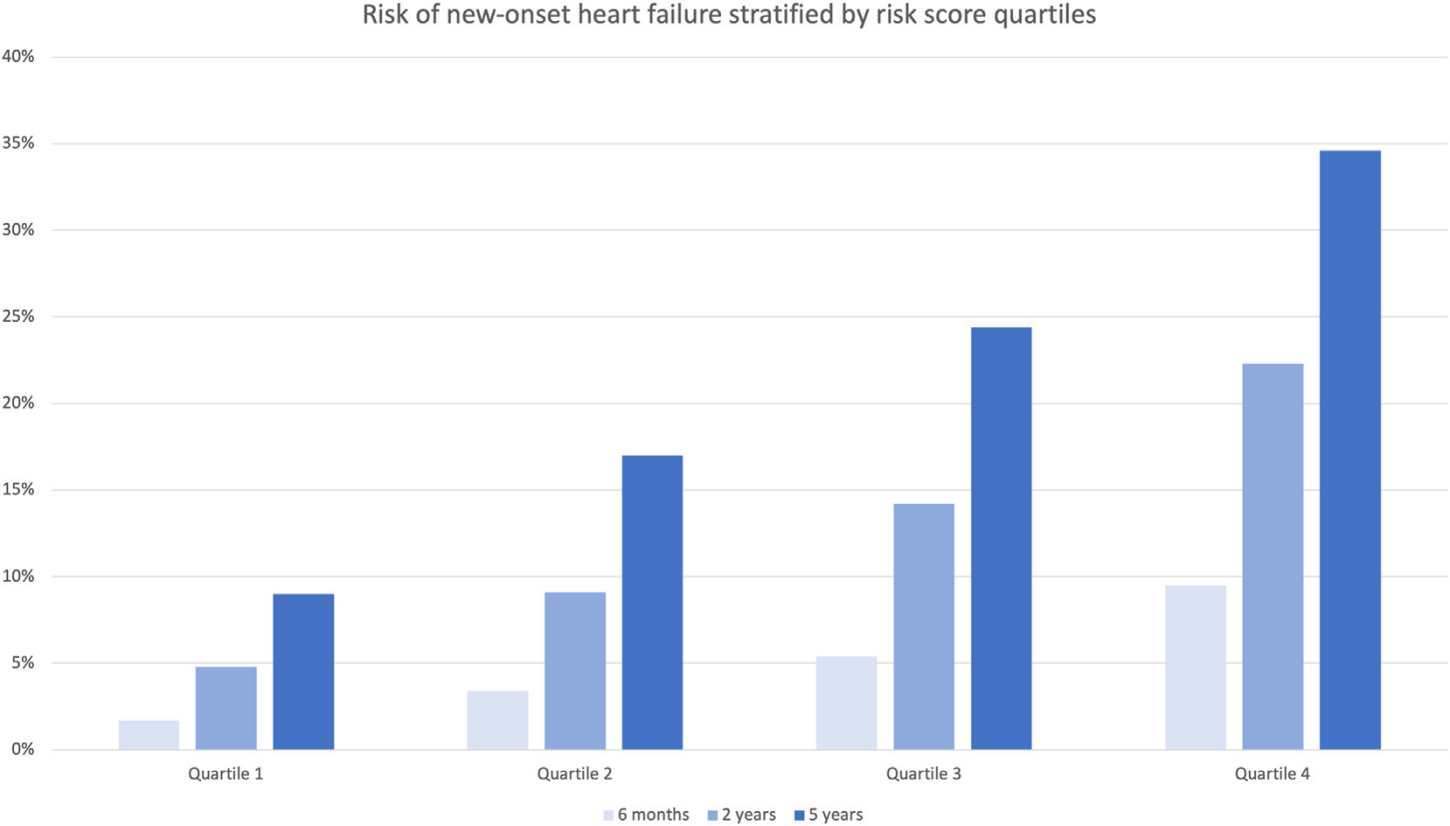
Risk of new-onset heart failure (in the validation set), based on risk score quartiles, after 6 months, 2 years, and 5 years. The x-axis shows quartile groups. The y-axis shows absolute risk of new-onset heart failure (in %).

**Figure 3:**
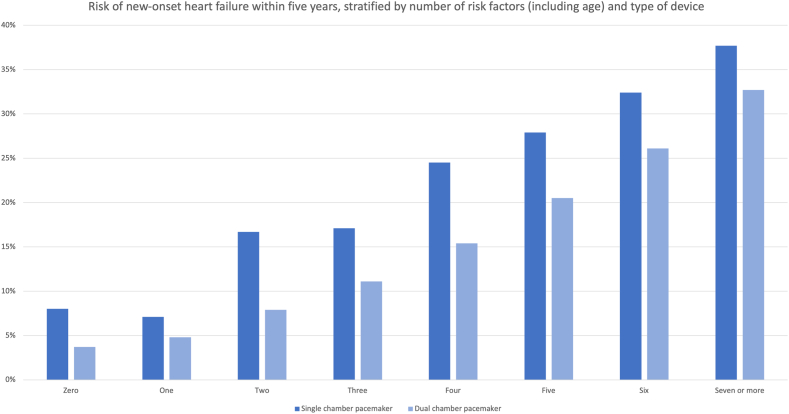
Risk of new-onset heart failure (in the entire cohort) at different time points postimplantation, stratified for number of risk factors and type of pacemaker.

The time when new-onset HF diagnosis occurred was evenly distributed between the first 6 months, 2 years, and up to 5 years postimplantation (Figure 2). Overall, the risk of new-onset HF was higher for single-chamber devices (32.7% vs 19.1%, *P* < .001), but patients with VVI devices also had a significantly higher risk score (*P* < .0001) and a much higher proportion of atrial fibrillation (82% vs 33%, *P* < .0001). In Figure 3, the incremental risk of HF is shown, based on number of risk factors (including age, 1 point per decile above 60 years) and stratified per dual- and single-chamber pacemakers.

## Discussion

In this nationwide cohort of all pacemaker implants in Sweden during a 15-year period, the observed overall risk for new-onset HF after implantation was over 20% within the first 5 years postimplantation. The risk was strongly associated with several well-known clinical risk factors, of which high age was the strongest independent predictor. Other strong risk factors included AV block or atrial fibrillation with slow conduction, likely representing a proxy for high percentage of RV pacing. This was strengthened by detailed data from a subset of the patients, indicating a strong association between both % RV pacing and HF, and with diagnosis of AV block or atrial fibrillation with slow conduction. However, there was still a substantial number of patients in the sick sinus syndrome group who were diagnosed with HF despite a presumed low burden of RV pacing. In fact, 30% of all patients who developed HF had this pacing indication, suggesting that other risk factors have a stronger influence on HF risk, compared with RV pacing alone. This is also in line with a previous RCT that did not find any difference in the risk of HF between AAIR and DDDR pacing modes in patients with sick sinus syndrome, nor any association to the % RV pacing in this patient group.^13^ The time of new-onset HF varied, with the majority of cases presenting within 2 years postimplantation, thus strengthening the temporal relationship with the pacemaker implant, even though a significant proportion of cases also occurred between 2 and 5 years postimplantation. The proposed risk score was an independent strong predictor of new-onset HF, and results were highly reproducible in the validation set of the cohort. The prognostic significance of a new-onset HF diagnosis was substantial, with the 5-year mortality increasing from 19.7% to 41.2%, and even more (to 48.3%) for those who were hospitalized due to HF.

Compared with a previous registry study from Denmark, the timing of new-onset HF was more spread out in our cohort, ranging from 6 months to 5 years after implantation. In the study by Tayal and colleagues,^6^ a comparison was made with an age- and gender-matched control group, which rendered information on relative risk increase for patients with RV pacing. The present study did not include a comparator group, but compared with the expected incidence of HF, there was a substantially higher risk at all 3 time points (6 months, 2 years, and 5 years), suggesting that the detrimental effects of RV pacing in patients with risk factors for HF accrue over time.^6^^,^^14^ In the study by Tayal and colleagues, new-onset HF within 30 days postimplantation was associated with the highest HR, but this could potentially have been an overestimation due to either previously undiagnosed HF or acute complications associated with the procedure, which later resolved. We chose to exclude the first 30 days (see reasoning in the Methods), which may result in a more accurate prediction of true new-onset HF after pacemaker implantation.

### Risk factors for new-onset HF

We identified 9 risk factors (including age) who had an independent association with new-onset HF: increasing age, male sex, atrial fibrillation, ischemic heart disease, diabetes, chronic kidney disease, hypertension, chronic obstructive lung disease, and AV block (with or without atrial fibrillation) as pacemaker indication. Risk of HF within 5 years ranged from 10% (lowest risk score quartile) to 32% (highest quartile). For comparison, standardized age adjusted incidence of HF in the general population >75 years of age was estimated at around 3% per year in a recent nationwide registry study.^14^ Our findings are in line with, and expand on, previous studies who have identified increasing age, renal failure, prior acute myocardial infarction, and atrial fibrillation as risk factors.^3^, ^4^, ^5^, ^6^^,^^15^ Furthermore, we show that chronic ischemic heart disease with or without prior myocardial infarction and chronic obstructive lung disease are also important risk factors. If the pacemaker indication was AV block or atrial fibrillation with slow ventricular rate (ie, expected high percentage of ventricular pacing), there was an association with higher risk of HF. This is in line with a previous registry-based study on 21202 patients from the United States, in which complete AV block was also associated with a higher HR for HF, particularly in the early phase within 6 months of implantation (adjusted HR 1.62, 95% CI 1.48–1.79, *P* < .001).^7^ The synergistic interaction and common risk factor sharing between chronic obstructive lung disease and HF is well known.^16^ It is therefore not surprising that obstructive lung disease is also a risk factor in the pacemaker-treated population, even though our study is the first to show this as an independent risk factor. In some previous studies, female sex has been identified as a strong protective factor, but in our cohort the relative risk increase for male sex was only 8%.

### Clinical implications

In patients with pacemaker, HF can occur as an independent event, related to traditional risk factors for HF only, such as hypertension and ischemic heart disease.^14^ However, adding RV pacing can potentially worsen the underlying condition, even if HF is unrelated to the pacing as such. For some patients, HF can also occur as a direct consequence of the RV pacing (ie, it would not have occurred if the patient did not receive RV pacing–induced dyssynchrony).

Given the higher mortality for patients with HF, it would be desirable to avoid RV pacing in all patients with ventricular dysfunction, regardless of the underlying cause. Avoidance of RV pacing is in many cases possible in patients in sinus node disease and even with AV block, and all manufacturers have built-in algorithms for doing so. In a recent prospective study from 2 similar cohorts, it was shown that use of AV hysteresis algorithms was associated with a reduction in the median ventricular pacing percentage, and in 1-year risk of HF hospitalization (58% relative risk reduction, *P* = .002).^17^ For patients with expected high percentage of RV pacing despite attempts to minimize RV pacing (ie, high-degree AV block), employing CSP or traditional CRT with a left ventricular lead could be an attractive alternative.^18^, ^19^, ^20^ In the current European Heart Rhythm Association consensus statement on CSP, the role of physiologic pacing to prevent pacing-induced HF is highlighted, supported by a growing number of prospective studies.^21^ However, choosing CRT or CSP as primary implant for all patients also entails drawbacks, as it adds complexity to the implantation procedure and also increases cost in the short term. Risk of lead-related dysfunction or complications are higher with CSP/CRT compared with conventional pacemakers, and long-term safety data on CSP are not yet available. Short- and medium-term data do suggest a higher risk of lead revision due to rising threshold or exit block in His bundle pacing, and a higher risk of lead fracture in left bundle branch area pacing.^18^^,^^22^ In the BIOPACE (Biventricular Pacing for Atrio-ventricular Block to Prevent Cardiac Desynchronization) study, choosing CRT to all-comers with AV block was not superior to RV pacing, even though there was a positive trend.^23^ This highlights the importance of patient selection, and the pivotal question is at which threshold level of potential/impeding HF risk would favor the use of a resynchronization device as primary implant. Our data suggest that for patients in the highest risk score quartile, or with ≥5 risk factors (including age), the 5-year risk of new-onset HF is 25% or higher. In reality, this number would be a little lower, as many patients in the highest age group will have an overall life expectancy that may favor a traditional pacemaker, regardless of risk score group. The financial cost varies between countries and health care systems, and prospective randomized trials are needed to evaluate total cost for the respective strategies. In doing this, it is also important to take into account the relatively high overall mortality. For patients with a high risk of dying from competing causes, it may not be justifiable or necessary to choose a resynchronization device, even if the predicted absolute risk of new-onset HF within 5 years is high. However, with the right threshold, using a primary resynchronization strategy may incur additional costs initially but prove to be the cheaper and prognostically better alternative for patients in the long run.

### Limitations

This was a nationwide cohort and follow-up was based on registry data, which means that individual data were ascertained via the medical records. This was a retrospective registry study, and hence no causal conclusions can be taken. There is a risk for misclassification of hospitalization data and outpatient data regarding the HF diagnosis. However, the Swedish national registries for disease diagnoses have previously been validated and shown to be very accurate.^11^ Due to the registry-based design, data on left ventricular ejection fraction and other echocardiography data were not available. Similarly, electrocardiography data, implant-related data, and follow-up device data (such as site of lead implantation, % RV pacing) were not available. During the time period for inclusion in this cohort, CSP was not used on a regular basis in Sweden, and there was no coding to identify the patients through the register. We estimate that the number of implants with CSP in the cohort is either nonexistent or negligible, and therefore will not have affected the results of the study.

## Conclusion

In this comprehensive national cohort, pacemaker therapy with a lead in the RV was associated with >20% risk of new-onset HF within 5 years, and patients experiencing new-onset HF had higher all-cause mortality. We identified 9 risk factors associated with the diagnosis of new-onset HF. If validated in prospective trials, the combined risk score based on these variables can potentially be used to identify patients at higher risk for new-onset HF after pacemaker implantation.

## References

[1] Glikson M., Nielsen J.C., Kronborg M.B. (2021). 2021 ESC guidelines on cardiac pacing and cardiac resynchronization therapy: developed by the Task Force on cardiac pacing and cardiac resynchronization therapy of the European Society of Cardiology (ESC) With the special contribution of the European Heart Rhythm Association (EHRA). Eur Heart J.

[2] Curtis A.B., Worley S.J., Adamson P.B., Biventricular versus Right Ventricular Pacing in Heart Failure Patients with Atrioventricular Block (BLOCK HF) Trial Investigators (2013). Biventricular pacing for atrioventricular block and systolic dysfunction. N Engl J Med.

[3] Kiehl E.L., Makki T., Kumar R. (2016). Incidence and predictors of right ventricular pacing-induced cardiomyopathy in patients with complete atrioventricular block and preserved left ventricular systolic function. Heart Rhythm.

[4] Sweeney M.O., Hellkamp A.S. (2006). Heart failure during cardiac pacing. Circulation.

[5] Khurshid S., Epstein A.E., Verdino R.J. (2014). Incidence and predictors of right ventricular pacing-induced cardiomyopathy. Heart Rhythm.

[6] Tayal B., Fruelund P., Sogaard P. (2019). Incidence of heart failure after pacemaker implantation: a nationwide Danish registry-based follow-up study. Eur Heart J.

[7] Merchant F.M., Hoskins M.H., Musat D.L. (2017). Incidence and time course for developing heart failure with high-burden right ventricular pacing. Circ Cardiovasc Qual Outcomes.

[8] Vassallo J.A., Cassidy D.M., Miller J.M., Buxton A.E., Marchlinski F.E., Josephson M.E. (1986). Left ventricular endocardial activation during right ventricular pacing: effect of underlying heart disease. J Am Coll Cardiol.

[9] Sweeney M.O., Hellkamp A.S., Ellenbogen K.A., MOST Investigators (2003). Adverse effect of ventricular pacing on heart failure and atrial fibrillation among patients with normal baseline QRS duration in a clinical trial of pacemaker therapy for sinus node dysfunction. Circulation.

[10] El-Chami M.F., Merchant F.M., Leon A.R. (2017). Leadless pacemakers. Am J Cardiol.

[11] Ludvigsson J.F., Otterblad-Olausson P., Pettersson B.U., Ekbom A. (2009). The Swedish personal identity number: possibilities and pitfalls in healthcare and medical research. Eur J Epidemiol.

[12] Ingelsson E., Arnlov J., Sundstrom J., Lind L. (2005). The validity of a diagnosis of heart failure in a hospital discharge register. Eur J Heart Fail.

[13] Riahi S., Nielsen J.C., Hjortshoj S. (2012). Heart failure in patients with sick sinus syndrome treated with single lead atrial or dual-chamber pacing: no association with pacing mode or right ventricular pacing site. Europace.

[14] Conrad N., Judge A., Tran J. (2018). Temporal trends and patterns in heart failure incidence: a population-based study of 4 million individuals. Lancet.

[15] Khazanie P., Hellkamp A.S., Fonarow G.C., Curtis L.H., Al-Khatib S.M., Hernandez A.F. (2018). Permanent pacemaker use among patients with heart failure and preserved ejection fraction: Findings from the Acute Decompensated Heart Failure National Registry (ADHERE) national registry. Am Heart J.

[16] Pellicori P., Cleland J.G.F., Clark A.L. (2020). Chronic obstructive pulmonary disease and heart failure: a breathless conspiracy. Heart Fail Clin.

[17] Arnold M., Richards M., D'Onofrio A. (2023). Avoiding unnecessary ventricular pacing is associated with reduced incidence of heart failure hospitalizations and persistent atrial fibrillation in pacemaker patients. Europace.

[18] Abdelrahman M., Subzposh F.A., Beer D. (2018). Clinical outcomes of his bundle pacing compared with right ventricular pacing. J Am Coll Cardiol.

[19] Vijayaraman P., Rajakumar C., Naperkowski A.M., Subzposh F.A. (2022). Clinical outcomes of left bundle branch area pacing compared with His bundle pacing. J Cardiovasc Electrophysiol.

[20] Qu Q., Sun J.Y., Zhang Z.Y. (2021). His-Purkinje conduction system pacing: A systematic review and network meta-analysis in bradycardia and conduction disorders. J Cardiovasc Electrophysiol.

[21] Burri H., Jastrzebski M., Cano O. (2023). EHRA clinical consensus statement on conduction system pacing implantation: executive summary. Endorsed by the Asia-Pacific Heart Rhythm Society (APHRS), Canadian Heart Rhythm Society (CHRS) and Latin-American Heart Rhythm Society (LAHRS). Europace.

[22] Ponnusamy S.S., Arora V., Namboodiri N., Kumar V., Kapoor A., Vijayaraman P. (2020). Left bundle branch pacing: a comprehensive review. J Cardiovasc Electrophysiol.

[23] Funck R.C., Mueller H.H., Lunati M., BioPace study group (2014). Characteristics of a large sample of candidates for permanent ventricular pacing included in the Biventricular Pacing for Atrio-ventricular Block to Prevent Cardiac Desynchronization Study (BioPace). Europace.

