# Supplementary material for: Risk factors and incidence of new-onset heart failure with conventional pacemaker implant: A nationwide study
Source: Heart Rhythm O2. 2024 Jul 23;5(9):623–30. doi: 10.1016/j.hroo.2024.07.012 (PMC11524952; doi:10.1016/j.hroo.2024.07.012)
Supplement: Supplemental tables [file mmc1.docx]

**Supplemental table S1.** Multivariable logistic regression analysis for prediction of new-onset heart failure in the entire cohort, excluding patients with an acute myocardial infarction between the date of implant and the date of new-onset heart failure diagnosis.

|  | **Multivariable analysis** | | |
| --- | --- | --- | --- |
|  | | **P-value** | **OR [95% CI]** |
| **Male sex** | |  |  |
| **Age** | |  |  |
| **-60** | | Ref |  |
| **60-69** | | <.001 | 1.29 [1.16-1.44] |
| **70-79** | | <.001 | 1.80 [1.63-1.99] |
| **80-89** | | <.001 | 2.65 [2.39-2.91] |
| **90-** | | <.001 | 3.01 [2.68-3.39] |
| **Atrial fibrillation (any)** | | <.001 | 1.65 [1.58-1.73] |
| **Ischemic heart disease (any)** | | <.001 | 1.59 [1.53-1.67] |
| **Diabetes (type I or type II)** | | <.001 | 1.31 [1.24-1.37] |
| **Chronic renal disease** | | <.001 | 1.56 [1.41-1.72] |
| **Chronic obstructive lung disease** | | <.001 | 1.66 [1.52-1.81] |
| **ECG indication (ref normal rhythm)** | | Ref |  |
| **AV block (I to III) or Atrial fibrillation** | | <.001 | 1.47 [1.38-1.55] |

**Supplemental table S2:**

Baseline demographic data for randomly selected cohort with detailed medical record data for QRS duration and percentage RV pacing.

|  | **Detailed cohort**  **(n=825)** |
| --- | --- |
| **Female sex** | 373 (45.2%) |
| **Age** | 77.9 [70.5-84.2] |
| **-60** | 64 (7.8%) |
| **60-69** | 129 (15.%) |
| **70-79** | 283 (34.3%) |
| **80-89** | 300 (36.4%) |
| **90-** | 48 (5.8%) |
| **Cerebrovascular disease** | 85 (10.3%) |
| **Atrial fibrillation** | 357 (43.3%) |
| **Any ischemic heart disease** | 247 (29.9%) |
| **IHD (chronic)** | 168 (20.4%) |
| **Prior AMI** | 80 (9.7%) |
| **Angina pectoris** | 128 (15.5%) |
| **Hypertension** | 483 (58.5%) |
| **Malignant tumour** | 227 (27.5%) |
| **Diabetes** | 125 (15.2%) |
| **Type II** | 123 (14.9%) |
| **Type I** | 22 (2.7%) |
| **Chronic liver disease** | 0 (0%) |
| **Chronic kidney disease** | 39 (4.7%) |
| **Sarcoidosis** | 6 (0.7%) |
| **COLD** | 37 (4.5%) |
| **ECG indication**  **Normal rhythm** | 5 (0.6%) |
| **AV block** | 383 (46.4%) |
| **Atrial fibrillation** | 99 (12.0%) |
| **Sick sinus syndrome** | 309 (37.5%) |
